# Supplementary material for: Treatment outcomes among young persons living with HIV who transitioned to adult care in southern Nigeria: A retrospective cohort study
Source: PLoS One. 2025 May 12;20(5):e0321962. doi: 10.1371/journal.pone.0321962 (PMC12068728; doi:10.1371/journal.pone.0321962)
Supplement: Attachment — Submitted filename: Response to Reviewers.docx [file pone.0321962.s003.docx]

18th January, 2025

The Editor,

PLOS ONE

**REBUTTAL LETTER**

Dear Editor,

Thank you for considering our manuscript for publication in PLUS ONE. We appreciate the detailed review and insightful comments provided by the reviewers. We have revised our manuscript to reflect the changes as suggested by the reviewers, and carefully provided a point-by-point response to all the comments. All modifications are highlighted in the manuscript using tracked changes. We are grateful for the opportunity to resubmit our revised manurscript and hope that it now meets PLOS ONE’s publication criteria. The point-by-point responses to the reviewer’s comments follow below.

**ACADEMIC EDITOR:**

***Financial disclosure:*** *As a secondary data analysis, the data is likely to have been collected from a program funded by an agency (refer to methods – study setting – line 133). Please indicate this as part of your financial disclosure. You should include the agreement/contract number and* *indicate that the findings are exclusively the opinions of the authors and do not represent the position of the funder.*

- **Authors’ Response:** We appreciate the editor's recommendation. However, this research did not receive specific funding, but we utilized data from a donor-funded project. We are unsure if this qualifies as a yes response to the financial disclosure question or if it's more appropriate to indicate that the authors received no specific funding for this work. We have revised the acknowledgement section to include the project funding source and agreement number as recommended. We have also indicated that the findings are exclusively the opinions of the authors and do not represent the position of the funder (lines 494-500).

***Data availability:*** *Not all data is presented within the manuscript. Therefore, indicate that data is available within the manuscript and other supporting documents, also provide rough data to support this as additional supporting document.*

- **Authors’ Response:** We thank the editor for this recommendation. We have included rough data as additional supporting document and modified the data availability statement to reflect that data is available within the manuscript and other supporting documents.

***Ethics statement:*** *You indicated N/A. However, from line 233 you reported “ethical approvals/exemptions”. Please clearly indicate your ethics statement and not N/A.*

- **Authors’ Response:** Thank you for highlighting this. The ethics statement has been added and now reads, "Permission to analyze the secondary program data was obtained from the Health Research Ethics Committee (HREC) in Akwa Ibom State on December 22, 2022 (HREC No. AKHREC/25/11/22/120), with an extension granted on January 9, 2024 (HREC No. AKHREC/5/5/23/149). Additional approval was obtained from the Office of International Research Ethics (OIRE) on February 24, 2023 (Project #: 2026278-1). Informed consent was waived because the study data was collected retrospectively, and only de-identified data were used for the analysis."

***Methods:***

- *Please clearly outline inclusion and exclusion criteria. though some of them have been mentioned under data collection, but there is need to explicitly outline them to avoid confusions. For example, the duration of the intervention; is it a client must be 1 year before data collection, or 8 months e.t.c.*
  - **Authors’ Response:** We thank the Editor for this recommendation. The inclusion and exclusion criteria have been outlined as follows: (lines 209-217)
    - Inclusion criteria
      - YLHIV who turned 25 years old in 2018 and 2021.
      - YLHIV enrolled in the peer-based clubs in the 2021 cohort
    - Exclusion criteria:
      - YLHIV who transferred out of the facility after transition were excluded because their treatment outcomes could not be established during the follow-up period.
      - YLHIV with less than one year of HIV treatment before the transition were excluded from the 2021 cohort due to their limited exposure to the intervention.
      - YLHIV not enrolled in the peer-based clubs in the 2021 cohort.
- *Line 203 – “transition was said to have happened after attaining 25 years”. Earlier it was stated that “transition assessment is conducted”. So, which of the two is more important? Attaining 25 years or passing transition assessment?*
  - **Authors’ Response:** We thank the Editor for this comment. Reaching 25 years of age was the most important criterion for transitioning. The transition assessment was informal and has been clarified in the manuscript. We have revised lines 192-194 to read “The second component was transition readiness assessment where healthcare workers routinely assessed YLHIV’s knowledge needs through informal discussions during clinic visits and club meetings and used this information to tailor support services.” Line 207-208 now reads “YLHIV were considered to have transitioned upon reaching the age of 25 years."
- *Is there transition assessment checklist? Please provide key questions in the checklist or provide it's picture as a figure.*
  - **Authors’ Response:** We thank the Editor for this question. There was no formal transition assessment checklist. Assessments were conducted informally as part of routine club meetings. This has been clarified in the manuscript (lines 192-194). "The second component was transition readiness assessment where healthcare workers routinely assessed YLHIV’s knowledge needs through informal discussions during clinic visits and club meetings and used this information to tailor support services."
- *Line 210 “retention in care disaggregated by….”. Retention in care is when someone satisfy the definition of “retention in care”. Consider using “continuity in care is disaggregated into….”.*
  - **Authors’ Response:** We appreciate this recommendation. “Retention in care” has been replaced with "continuity on treatment" throughout the manuscript.

**Results:**

- *Subsection “retention” – consider “continuity in care” instead. See earlier comment provided for line 210. Using “retention” the understanding is “client is retained”, and providing other categorization will cause confusion. However, retention can be a category under continuity in care, similarly retention with interruption, not retained, e.t.c can be other categories of continuity in care but not categories of retention.*
  - **Authors’ Response:** We appreciate this recommendation. The authors have revised the manuscript, and "continuity on treatment" has been used to replace "retention in care" throughout the manuscript.

**Acknowledgement:**

- *Consider acknowledging the funder.*
  - **Authors’ Response:** We appreciate the Editor for this recommendation. The authors have revised the acknowledgement section to include the funders. Lines 495-500 now reads, "We sincerely appreciate the peer supporters for their selfless service, the Akwa Ibom and Cross River State governments for providing a conducive environment for project implementation, and the generous funding and technical assistance provided by the American people through the US President’s Emergency Plan for AIDS Relief and the United States Agency for International Development."

**REVIEWER 1 COMMENTS:**

***Data presentation:***

1. *Line 270: 12 months percentages do not correlate with the data presented in Table 4. This is present in the abstract as well.*

- **Author’s Response:** We thank the reviewer for pointing out this discrepancy. The data has been corrected in all locations. Lines 60-63 of the abstract now read: "A higher proportion of those in the 2021 transition cohort was continuously retained both at 12 months and 24 months post-transitioning compared to those in the 2018 transition cohort (12 months: 96.7% vs 80.2%, p<0.001; 24 months: 92.7% vs 77.6% p<0.001)." The corrected data is also reflected in the manuscript on lines 290-293: "Also, a higher proportion of those in the 2021 transition cohort were continuously retained both at 12 months and 24 months post-transitioning compared to those in the 2018 transition cohort (12 months: 96.7% vs 80.2%, p<0.001; 24 months: 92.7% vs 77.6% p<0.001)."

2. *Line 276: retention at 12 months for females in 2018 percentage does not correlate with Table 5 data.*

- **Author’s Response:** We thank the reviewer for pointing out this discrepancy. The manuscript has been revised to ensure consistency between the in-text reporting and the table. Lines 297-299 now read: “As seen in Table 4, post-transition continuity on treatment at 12 months was higher among female YLHIV in the 2021 transition cohort compared to those in the 2018 transition cohort (97.4% vs 93.3%, OR: 2.71 95%CI [1.50 – 4.88]).”

3. *Table 7: Total number for column 12 months post-transition, 2018 transition year, number with undetectable VL noted as 197/323 – correct denominator is 328. Percentage calculated is correct for a denominator of 328.*
   - **Author’s Response:** We thank the reviewer for pointing out this discrepancy. The authors have revised the manuscript and Table 6 now reflects 328 as the denominator for the 2018 cohort. (line 340-341).
4. *Adherence support platform data, parameters and results not discussed in main body of manuscript. I suggest potentially renaming this table as the adherence support platform data does not necessarily fall under “demographic characteristics”, but this is in reference to the VL suppression through the post-transition period/ year of transition.*
   - **Author’s Response:** We appreciate the reviewer’s insightful comment regarding the adherence support platform data in Table 7. The data, parameters and results were not discussed in main body of manuscript because the authors did not intend to have the data presented. This is because we could not definitively establish client continuity on the adherence support platform over the 24-month period using the data available. This data has been removed from the table to ensure accuracy of the presented data.
5. *Line 136: suggest adding that the bracketed numbers are “n=” as this can be misinterpreted as a reference.*
   - **Author’s Response:** We appreciate the reviewers for this comment. We have revised the manuscript to address this. Line 148-149 now reads: “The study was conducted in 155 PEPFAR-supported health facilities which include 104 primary, 49 secondary and 2 tertiary health facilities.”
6. *Line 261 references Table 1 instead of Table 3 – requires a correction.*
   - **Author’s Response:** We thank the reviewer for identifying the incorrect table reference. We have corrected the reference to Table 2, as Table 1 was removed during this review process. The revised text on line 282 now reads: “This information is presented in Table 2.”
7. *Line 267 references Table 4 after a period – suggest that is is referenced more clearly.*
   - **Author’s Response:** We appreciate the reviewer for this observation. We have revised the text to adhere to PLOS ONE submission guidelines, ensuring clear referencing of tables within the text. The revised text now reads (lines 285-287): “As shown in Table 3, post-transition continuity on treatment at 12 months was higher for those in the 2021 transition cohort…”

**Technical clarifications:**

1. *I recommend changing the term “undetectable viraemia” to “undetectable viral load” which has been used in the body of the manuscript and better describes what the authors are referencing.*
   - **Author’s Response:** We appreciate the reviewer for this recommendation. We have implemented this change throughout the manuscript for consistency and clarity (lines 54, 64, 226, 231, 238, 320, 324, 332, 348).
2. *The Description of the Intervention (line 156) would be better linked to Table 2, and I would recommend removing Table 1 and incorporating this as descriptive text into the sub-headings of Pre-Intervention Period and Description of the Intervention.*
   - **Author’s Response:** We appreciate the reviewer for this recommendation. The authors accept this recommendation. Table 1 has been removed from the revised manuscript and the relevant components of Table 1 are incorporated in the Pre-intervention Period and Description of the Intervention sections.
3. *Table 2 has a bracketed number at the end of “Transfer to adult care/service provider”: (25). Suggest clarifying if this is the age at transfer of “25 years”. I recommend deleting if this is reference.*
   - **Author’s Response:** We appreciate the reviewer for this recommendation. We have included “years” as suggested to enhance clarity for the readers. The table now reads (Table 1), “Transfer to adult care/service provider (at 25 years).”

**References:**

1. *Vancouver referencing style is used throughout the relevant section, except for reference 3, 7, and 34; reference 50 appears to be incomplete.*
   - **Author’s Response:** We thank the reviewer for this comment. We have revised the reference section to ensure that all references in the manuscript conform with the Vancouver referencing style.
2. *Line 133 references the “2023 Spectrum Estimates”, but this is not included in the main references list.*
   - **Author’s Response:** We thank the reviewer for this comment. We have cited te source for the “2023 Spectrum Estimate” in the main reference list. It is now reference no. 23.

"Onovo AA, Adeyemi A, Onime D, Kalnoky M, Kagniniwa B, Dessie M, et al. Estimation of HIV prevalence and burden in Nigeria: a Bayesian predictive modelling study. EClinicalMedicine [Internet]. 2023 Aug 1 [cited 2025 Jan 4];62:102098. Available from: <https://pmc.ncbi.nlm.nih.gov/articles/PMC10393599/>"

3. *Spacing text from reference brackets would help in readability within the main body of the manuscript. This also includes adhering to one convention when referencing an author in text, such as for lines 372 – 373.*
   - **Author’s Response:** Thank you for your suggestion to improve readability. We have added spaces before the reference brackets throughout the manuscript. We have also standardized the in-text citation format for consistency. For example, the referenced text in lines 429 – 431 now reads: "For example, Meloni et al (14), Griffith et al (48) and Rungmaitree et al (49) reported 34.6%, 67% and 73.3% viral suppression at 12 months respectively (14,48,49)…”

**Typos, spelling, grammar and phrasing issues**

1. *I recommend maintaining formatting as per the PLOS guidelines – these have been followed, but not consistently, as seen with the indentation (lines 39 and 40, but not 56 – this should be the paragraph, not the heading), table formatting and referencing within the text (including location of table, table headings, and table footnotes), and heading formatting (font size for main vs. sub-heading).*
   - **Author’s Response:** We thank the reviewer for bringing the formatting inconsistency to our attention. The manuscript has been thoroughly reviewed and revised to ensure full adherence to PLOS ONE guidelines. This includes consistent indentation, correct table formatting (location, headings, and footnotes), and appropriate font sizes for main and sub-headings.
2. *This manuscript will benefit from thorough copyediting, as typographical errors are present throughout the text, including spelling (example: line 24), inconsistent capitalization of words (some examples: line 97 – in, Table 3 + line 388 for “dolutegravir, Table 1 – “trained”, etc.), maintaining standardized hyphenation throughout the manuscript body (12-month vs. 12 month), and switching of verb tense (present vs. past tense).*
   - **Author’s Response:** We appreciate the reviewer’s comment and we have carefully reviewed the entire text and corrected the typographical errors, ensured consistent capitalisation, standardised hyphenation and maintained uniform verb tense in the manuscript.
3. *Careful adherence to writing conventions needs to be maintained, such as numbering conventions at the start of sentences (line 146), and acronyms being expanded on first use in the text (see: ART, DSD).*
   - **Author’s Response:** We thank the reviewer for this recommendation. We have thoroughly revised the manuscript to adhere to writing conventions, ensuring that numbers are not used at the beginning of sentences and that all acronyms, such as ART and DSD, are expanded upon first use.
4. *Rewording confusing phrases such as lines 152 – 155 and lines 207 – 208.*
   - **Author’s Response:** We thank the reviewer for this recommendation. We have carefully revised the manuscript to enhance readability by rephrasing confusing phrases. For example, lines 152–154 now read: “Tertiary health facilities, which serve as the highest level of referrals, have highly experienced experts offering specialized care for disease conditions” and lines 203–204 now read: “YLHIV were considered to have transitioned upon reaching 25 years of age”.
5. *Line 239: recommend the use of “Informed” consent instead of “Ethical” consent.*
   - **Author’s Response:** We thank the reviewer for this recommendation, we have revised the ethics statement to replace "ethical consent" with "informed consent." (line 259) now reads: "Informed consent was waived because the study data was collected retrospectively, and only de-identified data were used for the analysis."

**REVIEWER 2 COMMENTS:**

**2.1.1. The Abstract**

*The abstract provides a clear and concise summary of the study, including the background, methods, results and conclusion. It could be improved by addressing the following:*

- *As the peer-based transition preparedness model is central to the study, the authors could consider adding a sentence in the introduction to provide more context about the model.*
  - **Author’s Response:** We appreciate the reviewer for this feedback. We have added a sentence in the introduction as recommended (lines 41-42). "In October 2019, a peer-based transition preparedness model was introduced as part of peer club activities to prepare young persons living with HIV (YLHIV) for adult care."
- *In the conclusion, the authors could highlight the potential implications of the findings for policy and practice to strengthen the impact of the conclusion.*
  - **Author’s Response:** We thank the reviewer for this recommendation. We have revised the conclusion to highlight policy implications (lines 69 -70) "Programs should implement targeted age-appropriate, peer-based interventions to address gaps in service delivery."

**2.1.2. Introduction**

*The introduction is well-written and provides a comprehensive overview of the context and significance of the study. It could however be enhanced by addressing the following:*

- *Provide more details on the peer-based transition preparedness model introduced in 2019, especially how it differs from previous models and why it was expected to improve outcomes considering the importance of transitioning YLHIV to adult care.*
  - **Author’s Response:** We thank the reviewer for this comment. We have expanded the introduction to include the specifics of the peer-based transition preparedness model (lines 100-103): "While existing programs primarily addressed treatment adherence and psychosocial support during adolescence, this model specifically prepared YLHIV for long-term adult care. It delivered peer-based services tailored age group, age-appropriate transition information, psychosocial support, and adherence support."
- *More recent studies or reviews could be added to support the need for the current research to better position the study within the existing body of knowledge.*
  - **Author’s Response:** We appreciate this feedback and have included more recent studies in the introduction to strengthen the rationale for our research (lines 104-114).
- *The study rationale is not mentioned explicitly. It would help to clearly state the gaps in the literature that this study aims to fill.*
  - **Author’s Response:** We appreciate this feedback. We have revised the manuscript to explicitly state the rationale and the gaps in the literature that our study is designed to address (lines 115-122): "While existing studies offer valuable insights into treatment outcomes for YLHIV transitioning to adult care, they have focused mainly on perinatally infected adolescents and often involve specialised or tertiary healthcare facilities. Consequently, there is a paucity of evidence on the post-transition outcomes for YLHIV in other facility types, such as primary and secondary care facilities, particularly in resource-limited settings like Nigeria."
- *The introduction does not explicitly state the objectives of the study. I would suggest that a clear statement of the study's objectives be explicitly added to provide a better roadmap for the reader.*
  - **Author’s Response:** We thank the reviewer for this observation. We have revised the manuscript by adding a clear statement of the study's objectives (lines 123-124). "The objective of this study was to assess the 12- and 24-month post-transition continuity of treatment and viral suppression rates among YLHIV transitioning to adult HIV care."

**2.1.3. Materials and Methods**

*The methods section is detailed and describes the study design, setting, population, and data collection procedures. The use of logistic regression analysis is appropriate for comparing retention in care and undetectable viraemia between the two groups. However, the section would benefit by the authors addressing the following:*

- *In addition to the authors stating the study design as a retrospective cohort study, they could consider adding that it is a before and after study. Furthermore, they could provide the rationale for choosing the study design to strengthen the justification.*
- **Author’s Response**: We thank the reviewer for this recommendation. We have updated the methods section to describe our study as a "retrospective before-and-after cohort study" (lines 131-132): "This was a retrospective before-and-after cohort study of YLHIV who transitioned to adult care.”We have also included a rationale for the design in lines 137-139: “The retrospective before-and-after cohort design was chosen because it allows for a direct comparison of outcomes to determine the effect of the intervention since this was part of program implementation."
- *Include a brief explanation of why the particular states were chosen. The authors also do not explicitly describe the sampling method for facilities and participants, and it could improve clarity if the manuscript explicitly states the sampling method used for both facilities and participants.*
- **Author’s Response:** We thank the reviewers for this feedback. We have revised the manuscript to include this. In lines 144-147, we say that “The study was conducted in Akwa Ibom and Cross River States. These states were selected for this study because they collectively account for 12% of Nigeria's national HIV burden (23) and received funding from the PEPFAR through USAID to implement the intervention”. Regarding the sampling method, the manuscript has also been updated to indicate that “All PEPFAR/USAID-supported facilities in these two states were included, and all YLHIV within these facilities who met the pre-specified inclusion criteria were included” (line 140-141).
- *The data collection process is described, but the researchers could clarify how missing data were handled and whether any imputation methods were used.*
- **Author’s Response:** The authors are thankful for this observation. The process for handling missing data has been updated appropriately. “All variables included in the logistic regression model had complete data, and no imputation methods were required.” (lines 247-248)
- *The statistical methods used are appropriate, but the authors could provide more details on the logistic regression models, including how potential confounders were handled.*
- **Author’s Response:** We thank the reviewer for the suggestion. We have expanded the description of the logistic regression models in the methods section to include details about the handling of potential confounders. The revised text now states: "The logistic regression model was adjusted for age at transition, sex, and health facility type. These potential confounders were identified based on prior literature and clinical relevance”. (lines 243-245)

**2.1.4. Results**
 *The results are presented clearly, with a focus on the key outcomes of retention in care and undetectable viraemia. The use of tables to summarize the characteristics of the study population and the outcomes is effective. The results section could benefit from the following revisions:*

- *More detailed descriptions of the statistical analyses performed, which could include specifying the statistical tests used to calculate the p-values, the logistic regression models used, together with explicitly stating the covariates included in the models.*
- **Author’s Response:** The authors are thankful for this observation. The authors have updated the data analysis description to include the use of the binary logistic regression model; and stated the test used as notes under each data table as well as in the data analysis section. The authors have also listed and described covariates used in the study.

- Generally, the results narrative uses p-values to indicate significance, but it would be helpful to also report the effect sizes and confidence intervals for the estimates, as they provide more information about the magnitude and precision of the effects.
- **Author’s Response:** The authors are thankful for this observation. The authors have updated the results’ reporting format to include both confidence intervals and effect sizes, providing a more comprehensive understanding of the magnitude and precision of the effects. This can be seen in Line 332-336: “The proportion of YLHIV with undetectable viral load was higher at 12 months in the 2021 transition cohort in all demographic categories except females (96.3% vs 60.9%, OR: 2.52, 95% CI [2.207 – 2.886]), and also at 24 months in all demographic categories except YLHIV receiving care in tertiary health facilities (85.1% vs 87.5%, OR 0.816, 95%CI [0.151 – 4.403]), compared to the 2018 year “Table 6”.”
- The retention rates at 12 and 24 months are reported using p-values. However, the odds ratios for retention at 24 months seem to suggest a lower retention rate for the 2021 cohort compared to the 2018 cohort, which contradicts the statement that a higher proportion of those who transitioned in 2021 were continuously retained. This discrepancy should be addressed.
- **Author’s Response:** The authors are thankful for this observation. While retention at 24 months was indeed lower in the 2021 cohort compared to the 2018 cohort, a higher proportion of individuals in the 2021 cohort were continuously retained on treatment without interruption over the 24-month period. We have revised the relevant section to clarify this distinction, as detailed on Lines 285-293: “Post-transition continuity on treatment at 12 months was higher for those in the 2021 transition cohort compared to those in the 2018 transitioned cohort (96.8% vs 93.6%, p=0.006). However, post-transition continuity on treatment at 24 months was lower for those in the 2021 transition cohort compared to those in the 2018 transition cohort (95.0% vs 99.7%, p=0.021) “Table 3”. Also, a higher proportion of those in the 2021 transition cohort were continuously retained both at 12 months and 24 months post-transitioning compared to those in the 2018 transition cohort (12 months: 96.7% vs 80.2%, p<0.001; 24 months: 92.7% vs 77.6% p<0.001).”

**2.1.5. Discussion**

The discussion interprets the findings in the context of the existing literature and the study's limitations. It provides a thoughtful analysis of the potential reasons for the observed improvements in treatment outcomes. It could however be strengthened by addressing the following:

- The authors could provide more details on the similarities and differences in study design, population and settings between their study and the cited studies to complement the comparison they have provided between their findings with those of the other studies.
- **Author’s Response:** We appreciate the reviewer for this recommendation. We have expanded the discussion to include comparisons with cited studies regarding design, population, and settings. An example can be seen on lines 370-376: “Our study shares a retrospective cohort design with Davies et al (13) and Meloni et al (14) but uniquely includes transitions across primary, secondary, and tertiary facilities in Nigeria. In the South Africa study, the majority of the transitions occurred before 15 years of age and involved a transition between healthcare facilities, whereas in our study the transitions to adult care were at 25 years of age and the majority of YLHIV remained at the same healthcare facility.”
- The discussion would be strengthened by a more detailed exploration of the potential mechanisms through which the peer-based transition preparedness model improves outcomes.
- **Author’s Response:** We appreciate the reviewer for this recommendation. The authors have added a section exploring how the peer-based model might improve outcomes (lines 377-381): “The success of the intervention in our study could be attributed to the peer-led support which enhanced psychosocial resilience and adherence education, which enabled a supportive environment for YLHIV during transition. The structured peer clubs and case management may have provided the tailored support that is crucial for maintaining high treatment retention settings.”
- Line 341 uses the word “disintermediation”. Since this word is not a common term in healthcare literature, it may be helpful to provide a brief explanation or definition of its use in the manuscript to ensure clarity for all readers, especially those who may not be familiar with its use in this context.
- **Author’s Response:** The authors are grateful to the reviewer for this observation. We have replaced the word "disintermediation" with a more elaborate explanation of the intended effect of YLHIV transition to adult care (lines 394-395): “This is a shift towards more direct, less mediated delivery models where YLHIV take on greater responsibility for their care.”

- The discussion on the higher retention rates at 12 months post-transition for the 2021 cohort is clear, but the lower retention rates at 24 months post-transition for the same cohort is not explained. The authors need to explore potential reasons for this decline in more detail.
- **Author’s Response:** We thank the reviewer for the insightful observation. We have expanded the discussion to explore potential reasons for the lower retention at 24 months in the 2021 transition cohort compared to the 2018 cohort (Lines 414-421): “This might be due to the impact of COVID-19 in 2020, which coincided with the second-year post-transition for the 2018 cohort. COVID-19 restrictions led to the scale-up of innovations like differentiated service delivery of ART including home refills, to sustain access to ART (27,41). Semo et al demonstrated that DSD improves client treatment retention, especially among adults, and the choice of a DSD model was influenced by clients’ experience, cost, and convenience (42). This effect of DSD may explain the higher treatment retention at 24 months among YLHIV in the 2018 cohort”.
- The authors have contextualized their findings within the broader literature on HIV care transitions but have not included a specific discussion on the unique challenges faced by YLHIV in Nigeria. This would provide more insight into the study’s results.
- **Author’s Response:** We appreciate the reviewer for the recommendation. We have added a discussion on the unique challenges YLHIV in Nigeria face. (lines 350-355): “In Nigeria, YLHIV face unique challenges such as stigma, lack of psychosocial support (especially among older adolescents who may be in boarding schools or residing in universities), financial stress, and disruptions in care due to the transition from paediatric to adult services, which can impact treatment adherence and outcomes.”
- Though the limitations of the study are acknowledged, the authors could also discuss the potential impact of these limitations on the study’s findings, e.g. how the use of secondary data might have influenced the results.
- **Author’s Response:** We appreciate the reviewer’s comment and have revised the limitations section to discuss how the limitations might have affected our results. Eg, (lines 455-457): "The use of secondary data limited our ability to consider other socioeconomic and clinical factors such as occupation and the presence of comorbidities, potentially skewing the outcomes by not capturing all influences on treatment adherence and retention."

**2.1.6. Conclusion**

The conclusion succinctly summarizes the main findings, and it appropriately emphasizes the positive impact of the peer-based transition preparedness model on treatment outcomes. It can however be strengthened by addressing a number of issues.

- More discussion can be provided on the implications of the findings for policy and practice, particularly in low- and middle-income countries.
- **Author’s Response:** We appreciate the reviewer’s recommendation. We have expanded our conclusion to discuss the policy implications of our findings (lines 480-486): “The evidence provided by this study will be useful to low and middle-income countries that provide ART services principally through primary and secondary healthcare facilities. Policymakers should consider implementing these tailored, age-specific interventions to bridge the gap in service delivery during the critical transition phase. This approach could help meet the UNAIDS targets for HIV control in resource-limited settings, where most YLHIV receive treatment.”
- The authors stated that “peer-led interventions to support YLHIV transitioning to adult care results in good treatment outcomes,” but it does not specify what these outcomes are. It would be more informative to mention the key outcomes, such as retention rates and viral suppression rates, directly in the conclusion.
- **Author’s Response:** We are grateful to the reviewers for this observation. The authors have revised the conclusion and specified the key outcomes measured in the study, which are continuity on treatment and undetectable viral load (lines 475-478): “Our study found that a peer-led intervention to support YLHIV transitioning to adult care was associated with good continuity on treatment and undetectable viral load at 12 and 24 months post-transition in relation to UNAIDS benchmarks.”
- The statement “irrespective of the facility type” is quite broad. While the study may have included various facility types, it’s important to note any differences in outcomes between these types, if they exist. The authors could conduct analyses to explain the differences e.g. stratifying the data by facility type, calculating outcomes by facility type, doing statistical analysis to compare the outcomes between the different facility types, adjusting for confounders, etc.
- **Author’s Response:** We thank the reviewer for this recommendation. The authors have highlighted the analyses on differences and similarities in outcomes across the facility types (primary, secondary and tertiary facilities throughout the manuscript. (eg. line 302-305): “With regards to facility type, continuity on treatment at 12 months was higher among YLHIV receiving care in primary health facilities in the 2021 transition cohort compared to those in the 2018 cohort (98.8% vs 92.6%, OR: 6.36, 95% CI [2.651 - 15.262]).” (lines 395-398): “YLHIV at primary healthcare facilities in our setting had a better retention at 12 months after the intervention compared to before, although the retention rate at 24 months was comparable before and after the intervention in this health facility type.”
- The conclusion does not suggest any future research directions. It would be beneficial for the authors to highlight areas where further investigation is needed, especially given the limitations mentioned in the discussion.
- **Author’s Response:** We have added recommendations for future research (lines 486-488): “Further investigation is needed to assess longer-term outcomes (up to 60 months) and to consider socioeconomic factors and the perspectives of YLHIV and healthcare workers on the transition preparedness model.”

**2.2. Minor Issues

2.2.1. Materials and Methods**

- Though the peer-based transition preparedness model is provided in table 2, it could be helpful if the authors included a brief summary in the text.
- **Author’s Response:** We have included a summary of the peer-based transition preparedness model in the text to complement the information in Table 1. Lines 189-202: “The peer-based transition preparedness model had four essential components. First was orienting YLHIV on the age of transition. This was done from the age of 15 years. YLHIV received orientation at club meetings about the transition to adult care at 25 years of age. The second component was transition readiness assessment where healthcare workers routinely assessed YLHIV’s knowledge needs through informal discussions during clinic visits and club meetings and used this information to tailor support services. The third component was transition preparedness using a six-month transition curriculum. This curriculum covered topics on self-care, roles, responsibilities, expectations during and after the transition, treatment literacy, sexual and reproductive health, disclosure to partners, and peer support. Routine refreshers were provided at age-band groupings. The fourth was the actual transfer to adult care, where YLHIV were disengaged from club activities and were assigned/physically introduced to adult case managers when they turned 25 years old. They were also linked to Differentiated service delivery (DSD) for adults”

- The authors mention ethical approval was obtained. It would help if they could discuss the ethical considerations in the methods section.
- **Author’s Response:** The authors appreciate this recommendation. The ethical statement has been revised to discuss key components of the ethical approval.

Specifically, we have detailed the process for obtaining ethical approval, the measures taken to ensure participant confidentiality, and how informed consent was managed, especially given the use of secondary data (Line 254 -261).

**2.2.2. Results**

- The viral load suppression data is well-presented in Table 6. I would suggest that the authors consider adding a note to explain the categories of viral load changes over the 24 months post-transitioning.
- **Author’s Response:** We thank the reviewer for this recommendation. We have included the categories of viral load changes as footnotes to Table 5 to improve clarity (lines 328-331): “*Denominator includes only those with viral load results within the period; **only for those with at least two viral load results post-transition over 24 months; 2018 = 202; 2021 = 1110. Sustained undetectable viral load means viral load ≤50 copies/ml, low-level viral load means ≥1 viral load between 51 and 999 copies/ml, and high viral load means any viral load ≥1000 copies/ml during the period.”

**2.2.8. Language and Grammar**

- The language and grammar of the manuscript are generally clear and understandable. However, there are several areas where the language can be improved for clarity, conciseness, and readability. For example: "Thesa" instead of "These" in line 23.
- **Author’s Response:** We thank the reviewer for this feedback, we have corrected this typographical error in the manuscript (line 25): “¶These authors contributed equally to this work.”
- The sentence starting in line 62, “Undetectable viraemia was higher among the 2021 cohort ….” Could be rephrased.
- **Author’s Response:** We thank the reviewer for this recommendation. We have rephrased this sentence for clarity (lines 63-65): "Similarly, the proportion of YLHIV with undetectable viral load in the 2021 cohort was significantly higher than those in the 2018 cohort at both 12 months (96.1% vs 60.1%, p<0.001) and 24 months (93.3% vs 80.6%, p<0.001) respectively."
- Some long sentences could be broken down for better readability. E.g. the sentence starting "YLHIV is a heterogeneous group that includes adolescents (10-19 years) and youth (15-24 years) with both ….”
- **Author’s Response:** We thank the reviewer for this feedback, we have broken down long sentences for better readability. Eg the referenced sentence (lines 81-83) now reads: "YLHIV (adolescents aged 10-19 and youth aged 15-24) face a critical period when transitioning from adolescent to adult HIV care. This transition often coincides with significant physiological, psychological, and social changes."
- It would help to use simpler language and words, and avoid uncommon words like “disintermediation”.
- **Author’s Response:** We thank the reviewer for this comment. We have simplified our language as much as possible throughout the manuscript. The word "disintermediation" has been replaced with a clearer description in lines 394-395: “This is a shift towards more direct, less mediated delivery models where YLHIV take on greater responsibility for their care .”
- A thorough proofread of the manuscript could help identify and correct grammatical errors and typos, and improve some of the language for better readability and flow.
- **Author’s Response:** The authors appreciate this feedback and have undertaken a thorough proofreading of the manuscript to correct grammatical and typographical errors.
