# Supplementary material for: Treatment outcomes among young persons living with HIV who transitioned to adult care in southern Nigeria: A retrospective cohort study
Source: PLoS One. 2025 May 12;20(5):e0321962. doi: 10.1371/journal.pone.0321962 (PMC12068728; doi:10.1371/journal.pone.0321962)
Supplement: Attachment — Submitted filename: Response_to_Reviewers_auresp_2.docx [file pone.0321962.s004.docx]

12th March 2025

The Editor,

PLOS ONE

**REBUTTAL LETTER**

Dear Editor,

Thank you for considering our manuscript for publication in PLOS ONE. We appreciate the feedback from the reviewers and the recommendation of the editor. We have revised our manuscript to reflect the changes as suggested by the editor and carefully responded to the outstanding comment. The revisions are highlighted in the manuscript using tracked changes. We are grateful for the opportunity to resubmit our revised manuscript and are hopeful that it now meets PLOS ONE’s publication criteria. The point-by-point responses to the editor’s comment is below.

**ACADEMIC EDITOR:**

However, your funding statement is still not acceptable. If fund have been allocated for the program that resulted in the data used for this study, then the study is considered funded. The funder does not necessarily need to participate or directly fund this study (manuscript). Therefore, we invite you to submit revised funding statement indicating the funder, the contract/agreement number. Also adjust your acknowledgement by removing any information about the funder.

- **Authors’ Response:** We thank the editor for this recommendation. We have included the funder and the project agreement number in the cover letter as guided by the Straive Editorial Assistant of the journal. We have also removed all information about the funder in the acknowledgement.
